# Observational study on the prognostic value of testosterone and adiposity in postmenopausal estrogen receptor positive breast cancer patients

**DOI:** 10.1186/s12885-018-4558-4

**Published:** 2018-06-13

**Authors:** Elisabetta Venturelli, Annalisa Orenti, Aline S. C. Fabricio, Giulia Garrone, Roberto Agresti, Biagio Paolini, Chiara Bonini, Massimo Gion, Franco Berrino, Christine Desmedt, Danila Coradini, Elia Biganzoli

**Affiliations:** 1 Department of Research, Fondazione IRCCS Istituto Nazionale dei Tumori, Via Venezian 1, 20133 Milan, Italy; 2 Laboratory of Medical Statistics and Epidemiology,“Giulio A. Maccacaro”, Department of Clinical Sciences and Community Health, University of Milan, Via Vanzetti 5, 20133 Milan, Italy; 3 Regional Center for Biomarkers, Department of Clinical Pathology and Transfusion Medicine, Azienda ULSS3 Serenissima, Regional Hospital, Campo SS Giovanni e Paolo 6777, 30122 Venice, Italy; 4 Breast Surgery Unit, Fondazione IRCCS Istituto Nazionale dei Tumori, Via Venezian 1, 20133 Milan, Italy; 5 Department of Diagnostic Pathology and Laboratory, Fondazione IRCCS Istituto Nazionale dei Tumori, Via Venezian 1, 20133 Milan, Italy; 6 Breast Cancer Translational Research Laboratory, J. C. Heuson, Institut Jules Bordet, Université Libre de Bruxelles, 121 Boulevard de Waterloo, 1000 Bruxelles, Brussels Belgium; 7 Unit of Medical Statistics, Biometry and Bioinformatics, Campus Cascina Rosa, Fondazione IRCCS Istituto Nazionale Tumori, Via Vanzetti 5, 20133 Milan, Italy

**Keywords:** Testosterone, Body mass index, Estrogen receptor, Postmenopausal women, Breast cancer

## Abstract

**Background:**

Despite the clear endocrine-metabolic relationship between androgenic activity and adiposity, the role of androgens in breast cancer prognosis according to patient’s adiposity is scarcely explored. Here, we aimed at investigating the prognostic value of circulating testosterone in association with patient’s body mass index (BMI).

**Methods:**

Circulating testosterone and BMI were evaluated at breast cancer diagnosis in 460 estrogen receptor (ER)-positive postmenopausal patients. Local relapse, distant metastasi(e)s and contralateral breast cancer were considered recurrence events. The Kruskal-Wallis test was performed to evaluate if testosterone levels differed within subgroups of categorical tumour characteristics. The Cox proportional hazard regression model was fitted to estimate the impact of standard prognostic factors on relapse-specific hazard ratio (HR). After backward selection, a model including continuous testosterone level, BMI categories (< 25, normal-weight; =25–30, overweight; ≥30 kg/m^2^, obese), tumour size and lymph nodes number was fitted. Furthermore, Cox models provided the relapse-specific HRs for median, third quartile and 95th percentile compared to the first quartile of testosterone levels, stratified by BMI categories.

**Results:**

During a median follow up of 6.3 years, 45 patients relapsed. Testosterone levels significantly increased across BMI categories (*p* = 0.001). Both circulating testosterone and BMI were positively associated with disease free survival (*p* = 0.005 and *p* = 0.021, respectively). A significant interaction was found between testosterone and BMI (*p* = 0.006). For normal-weight women, testosterone concentration around median (0.403 ng/mL) or third quartile (0.532 ng/mL) showed a high significant HR of relapse (5.52; 95% CI:1.65–18.49 and 4.55; 95% CI:1.09–18.98, respectively). Overweight patients showed increased HR at increasing testosterone levels, reaching a significant high HR (4.68; 95% CI:1.39–15.70) for testosterone values of 0.782 ng/mL (95th percentile). For obese patients HR decreased (not significantly) at increased testosterone concentrations, explaining the interaction between testosterone levels and BMI categories.

**Conclusions:**

In ER-positive postmenopausal breast cancer patients, high testosterone levels are associated with worse prognosis in normal-weight and overweight women, whereas in obese seems to be associated with a better outcome. Although the results require further validation, they suggest that assessment of circulating testosterone and BMI could help to identify postmenopausal ER-positive patients at higher risk of relapse and potentially open new therapeutic strategies.

**Electronic supplementary material:**

The online version of this article (10.1186/s12885-018-4558-4) contains supplementary material, which is available to authorized users.

## Background

The role of androgens in breast cancer is an old debated topic. While many aspects still remain unclear, several studies consistently demonstrated that high levels of testosterone are associated with an increased risk of developing breast cancer, especially in ER-positive tumours [1–7]. Conversely, few studies have investigated the potential prognostic value of testosterone [8–11]. In particular, previous studies from our group showed that elevated urinary levels of testosterone were associated with a worse outcome [8]. More recently, prospective studies have provided evidence for an increased rate of progression in postmenopausal patients with high circulating levels of testosterone [9–11]. It is known that, in postmenopausal women, adiposity ― especially abdominal fatness ― is associated with high levels of circulating testosterone and estradiol. A positive association has also been described between sex hormones levels and Body Mass Index (BMI), the parameter used as proxy for adiposity [12–14]. Increased BMI has, in turn, been associated with both onset and progression of postmenopausal breast cancer patients in particular with ER-positive tumours [15–17]. After menopause, with the decline of the ovarian production of estrogens and with the tendency of women to gain weight, a concomitant presence of high androgenic activity can trigger a series of endocrine-metabolic disorders including sex hormones imbalance, insulin resistance, metabolic syndrome and inflammatory response [18, 19], possibly providing a suitable milieu for the growth of breast cancer [20]. Several evidences suggest the presence of a coordinate mechanism whereby, when estrogens lessen, testosterone promotes a redistribution of fat deposits that preferentially accumulate in the abdomen [21, 22]. This excess of visceral fat, known as central obesity, plays a relevant role in favoring the onset of insulin resistance and related dismetabolism, such as hyperinsulinemia, IGF-I hyper-production and metabolic syndrome [18, 19]. An excessive insulin production also stimulates the synthesis of androgens by the ovary and inhibits the hepatic production of sex hormone-binding globulin (SHBG), the protein that carries sex hormones through the bloodstream [23, 24]. The result is a further increase in total and free testosterone levels, which in turn, on the one hand favor higher estradiol bioavailability by its aromatization at adipose tissue levels [25] and by binding SHBG with a greater affinity than estrogens [26], and on the other hand strengthen the progression of insulin resistance by continuing to promote the visceral fat accumulation [21, 22]. This endocrine-metabolic loop favored by the advent of menopause, may particularly be harmful for recurrences of hormone-dependent breast cancer, i.e., tumours expressing ER, since the high availability of sex hormones and growth factors acts as a strong stimulus for tumour growth [27, 28].

Despite the clear interrelationship between adiposity and androgenic activity, studies specifically aimed at investigating the impact of this relationship on prognosis of postmenopausal ER-positive breast cancer patients are lacking. Therefore, in the present observational retrospective study we investigated the prognostic role of testosterone circulating levels as a function of patient’s BMI, in an institutional series of consecutive postmenopausal women with ER-positive tumours.

## Methods

### Patients

This was an observational retrospective study focusing on the first consecutive 592 eligible postmenopausal patients recruited at Fondazione IRCSS Istituto Nazionale Tumori of Milan (INT) from December 2003 to December 2006 in the TPM (Testosterone Prognosis, Mammary cancer) cohort and whose follow-up ended at December 2012. The study was approved by the INT Scientific and Ethical Committee and written informed consent was obtained from all included patients. Clinical and pathological information (self-reported weight and height- recorded during first clinic visit), date and type of surgery, histology, TNM stage, tumour grade, immuno-histochemical evaluation of ER and Progesterone Receptor (PR) status, and HER2 overexpression), as well as follow-up information (radiotherapy, adjuvant therapy, date of last control, date and site of breast cancer recurrence, presence of a primary non-breast cancer, life-status, and date and cause of death), obtained from medical records, were retrieved from the TPM database. Extensively presented elsewhere [14, 29, 30], the whole TPM cohort includes patients with a primary breast cancer surgically treated at the Breast Surgery Unit of the INT from December 2003 to March 2011. Inclusion criterion was having histologically confirmed non-metastatic breast carcinoma (any T, any N, and M0). Exclusion criteria included the presence of a nonepithelial cancer, a previous cancer diagnosis (except for in situ cervical cancer or non-melanoma skin cancer), and the treatment with neoadjuvant chemo- or hormone therapy. Before surgery, patients provided a fasting blood sample which was processed to obtain serum, subdivided into aliquots and stored at − 80 °C until use.

The expression of androgen receptor (AR) in tumour tissue was evaluated by tissue microarrays as described elsewhere [30].

TPM-postmenopausal patients were treated according to international guidelines for breast cancer management [31]. Subjects were routinely followed-up from the entry to the study to any of the following events: breast cancer recurrence, other non-breast primary cancer, death or scheduled end of follow-up. Information about vital status of patients who had discontinued their regular control at our institution was obtained through telephone interviews with patients or their next of kin. Interviews were carried out by expert trained personnel in accordance with a predefined protocol. Of the 592 initial postmenopausal breast cancer women recruited in the TPM cohort, 37 were excluded because they proved to fall outside the recruitment criteria, 19 violated postmenopausal criteria (last menstruation ≥12 months before enrolment, bilateral oophorectomy or hysterectomy without oophorectomy or monolateral oophorectomy and ≥ 50 y old and estradiol ≤30 pg/mL); 10 were excluded because they stopped hormone replacement therapy only 3 months before recruitment; 5 had previous cancer diagnosis; 1 had already metastatic disease and 2 received neoadjuvant therapy. Of the 555 remaining in the cohort, 460 were identified as ER-positive and represent the current study population (Additional file 1: Figure S1).

### Testosterone assay

Baseline serum testosterone levels were evaluated in duplicate using RIA commercial kits (Orion Diagnostica, Espoo, Finland) according to the manufacturer’s instructions. The kit detection limit was 0.03 ng/mL. Interassay coefficients of variation were 6.4 and 7.6% for mean testosterone titers of 0.359 and 0.455 ng/mL, respectively.

### Statistical analysis

According to WHO recommendation (Report of a WHO Expert Committee 1995) BMI was classified as < 25 (normal weight), =25–30 (overweight) and ≥ 30 kg/m^2^ (obese). Only 5 women were mild underweight and were included in the first BMI category. Tumour size was classified according to pT categories, as pT1, pT2 and pT3-pT4, tumour histology as IDC (Invasive Ductal Carcinoma), ILC (Invasive Lobular Carcinoma), mixed IDC/ILC and other histology; tumour grade as (G)1, G2 and G3; the number of metastatic axillary lymph nodes (N) as =0, 1–2 and > 2.

Steroid receptor status was defined as positive when the percentage of stained tumour cells was ≥10%, ≥10% and ≥ 1% for ER, PR and AR, respectively [30]. Categorical tumour characteristics were summarized by means of counts and percentages and chi-square test was performed to evaluate if tumour characteristics differed according to BMI categories. To assess if testosterone levels differed within subgroups of categorical variables, boxplots of testosterone levels according the categorized variables were drawn and non-parametric Kruskal-Wallis tests were performed. Time from surgery to first relapse was used in the estimation of disease free survival (DFS). The events used as end points in the determination of DFS included first local recurrence of disease, axillary and distant metastases, contralateral breast cancer. Patients who died without experiencing a breast cancer relapse previously were censored in the analysis of DFS.

Cox proportional hazard regression model was fitted to estimate the impact of different standard breast cancer prognostic factors as covariates on relapse-specific hazard. The initial model included steroid hormone status (PR and AR), tumour characteristics (histology, size, grade, number of metastatic lymph nodes and HER2 status), and patient characteristics (age, testosterone level, and BMI categories) as independent covariates. The proportional hazard assumption of the Cox model was also assessed. First, models were developed to identify variables most predictive of events, using a stepwise approach employing the most significant predictors. After backward selection, a model including only testosterone level as a continuous variable, BMI category, tumour size and the number of metastatic lymph nodes was fitted. To better interpolate smoothing effect of continuous covariates, nonlinear effect of testosterone was modeled by means of a cubic restricted spline with 3 knots [32]. The interaction between testosterone and BMI categories was considered to model the differential testosterone effect over different BMI classes. Thus, the final Cox model included continuous testosterone level (using a cubic spline with 3 knots), categorical BMI, the interaction term between testosterone and BMI, and tumour size and metastatic axillary lymph as adjusting covariates.

A plot of testosterone relapse-specific hazard ratio (HR) in the different BMI categories was drawn; the first quartile of circulating testosterone in the whole sample was taken as reference value. Accordingly, within the different BMI categories, numerical estimates of relapse-specific HRs were provided for three values of testosterone level, corresponding to median, third quartile and 95th percentile with respect to the first quartile of testosterone levels. Pertinent 95% confidence intervals were also provided. All *p*-values refer to two-sided statistical tests with *p* < 0.05 considered statistically significant. The analyses were performed using R statistical software v.3.3.2 with *survival* and *rms* packages.

## Results

### Serum testosterone and tumour characteristics

**Fig. 1:**
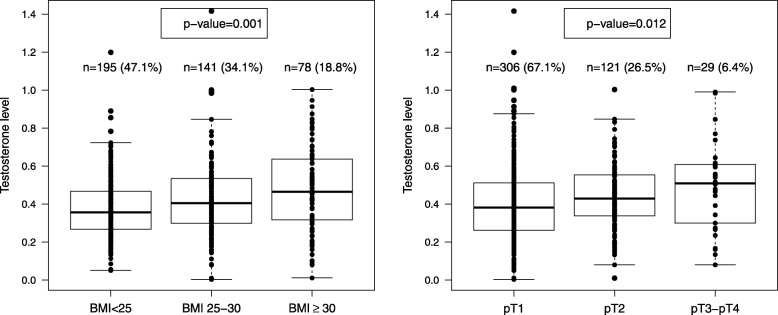
Boxplots of circulating levels of testosterone (ng/mL) according to body mass index (BMI) and tumour size (pT) of ER-positive postmenopausal breast cancer patients Number and percentage of patients in each group are reported and *p*-values are given. The bar inside the box is the median value and the box upper and lower dimensions define the inter-quartile range.

Table 1 shows the baseline characteristics of the 460 ER-positive postmenopausal women as a whole and according to BMI categories. Out of 460 ER-positive patients, 195 women had BMI < 25, 141 BMI = 25–30, 78 with BMI ≥ 30, and 46 unknown BMI. The median value of testosterone in serum of ER-positive patients was 0.403 ng/mL (1st and 3rd quartile: 0.278, 0.532 ng/mL). The associations between testosterone levels and the tumour characteristics found in the original 592 TPM-postmenopausal women have been extensively described in three previously published studies [14, 29, 30], and were substantially confirmed in the current series of 460 ER-positive patients. In particular, it was confirmed that testosterone levels significantly increased across BMI categories (*p* = 0.001; Fig. 1). Significant increase in the levels of the androgen was also confirmed for increasing tumours size (*p* = 0.012). No significant associations were found for the remaining tumour characteristics (histology, tumour grade, axillary nodal status, AR, PR and HER2 status; Additional file 2: Figure S2).

**Table 1:** Patient and tumour characteristics of ER-positive postmenopausal breast cancer patients as whole and according to BMI categories

	Total cases *N* = 460	BMI < 25 *N* = 195	BMI 25–30 *N* = 141	BMI ≥30 *N* = 78	*p*-value^a^
Histology					
IDC	350 (76.9%)	135 (71.1%)	113 (80.1%)	65 (83.3%)	0.132
IDC + ILC	42 (9.2%)	19 (10.0%)	12 (8.5%)	6 (7.7%)	
ILC	63 (13.8%)	36 (18.9%)	16 (11.3%)	7 (9.0%)	
Other	5	5	0	0	
Tumour size					
pT1	306 (67.1%)	137 (71.7%)	92 (65.2%)	46 (59.0%)	0.167
pT2	121 (26.5%)	41 (21.5%)	42 (29.8%)	24 (30.8%)	
pT3-pT4	29 (6.4%)	13 (6.8%)	7 (5.0%)	8 (10.3%)	
NA	4	4	0	0	
Tumour grade					
G1	35 (7.7%)	18 (9.4%)	9 (6.4%)	4 (5.1%)	0.166
G2	293 (64.4%)	129 (67.2%)	82 (58.6%)	51 (65.4%)	
G3	127 (27.9%)	45 (23.4%)	49 (35.0%)	23 (29.5%)	
NA	5	3	1	0	
Nodal status					
Negative	288 (64.3%)	122 (64.2%)	83 (61.5%)	44 (57.1%)	0.689
Positive (1–2)	88 (19.6%)	34 (17.9%)	31 (23.0%)	18 (23.4%)	
Positive (> 2)	72 (16.1%)	34 (17.9%)	21 (15.6%)	15 (19.5%)	
NA	12	5	6	1	
Progesterone receptor status					
Negative	103 (22.4%)	52 (26.7%)	24 (17.0%)	11 (14.1%)	0.025
Positive	357 (77.6%)	143 (73.3%)	117 (83.0%)	67 (85.9%)	
Androgen receptor status					
Negative	37 (8.5%)	18 (9.9%)	15 (10.9%)	2 (2.7%)	0.099
Positive < 60%	162 (37.1%)	70 (38.5%)	50 (36.5%)	22 (29.7%)	
Positive ≥60%	238 (54.5%)	94 (51.6%)	72 (52.6%)	50 (67.6%)	
NA	23	13	4	4	
HER2 status					
Negative	176 (55.5%)	78 (56.5%)	51 (51.0%)	27 (56.2%)	0.520
Positive 2+	90 (28.4%)	42 (30.4%)	28 (28.0%)	15 (31.2%)	
Positive 3+	51 (16.1%)	18 (13%)	21 (21.0%)	6 (12.5%)	
NA	143	57	41	30	
Endocrine therapy					
No	30 (6.5%)	14 (7.2%)	6 (4.3%)	5 (6.4%)	0.543
Yes	429 (93.5%)	181 (92.8%)	134 (95.7%)	73 (93.6%)	
NA	1	0	1	0	

^a^All *p*-values were evaluated excluding NA category or Other category for Histology variable

*ER* Estrogen Receptor, *IDC* Invasive Ductal Carcinoma, *ILC* Invasive Lobular Carcinoma, *NA* Not Available data

### Serum testosterone, BMI, and disease-free survival

During a median follow up of 6.3 years (interquartile range = 5.5–7.3 years), 45 patients experienced breast cancer recurrence within 7 years of follow-up. In particular, relapse occurred in 18 women with BMI < 25, 14 with BMI 25–30, 11 with BMI ≥ 30, and 2 with unknown BMI. Out of 460 ER-positive postmenopausal women, 31 died: 8 because of progressive disease and 23 for other causes than cancer. Patients who died of breast cancer and previously experienced a cancer relapse were already included as events in DFS.

Fitting the final Cox model, it emerged that relapse-specific hazard was significantly associated with circulating levels of testosterone (*p* = 0.005), the latter having a significant nonlinear effect (*p* = 0.042), as well as with BMI (*p* = 0.021), as expected, with metastatic axillary lymph nodes (*p* = 0.046), and with tumour size, even though non significantly (*p* = 0.061). Moreover, the Cox model showed that there is a significant interaction between testosterone levels and BMI categories (*p* = 0.006), indicating a different impact of testosterone level on DFS and consequently on relapse-specific hazard, among the three BMI categories.

Considering the significant interaction between testosterone levels and BMI, relapse specific hazard as a function of continuous androgen levels have to be considered separately for the three BMI groups.

**Fig. 2:**
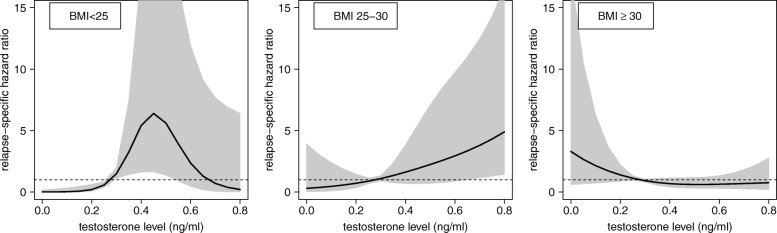
Estimated effect of circulating levels of testosterone on breast cancer relapse in the different BMI groups of ER-positive postmenopausal breast cancer patients according to Cox proportional hazard regression model for DFS, using testosterone levels as a continuous variable, BMI category, interaction terms between testosterone and BMI, with tumour size and number of metastatic lymph nodes as adjusting covariates. Relapse-specific hazard ratios with pertinent 95% confidence intervals are plotted. First quartile of testosterone level (0.278 ng/mL) is the reference value

The corresponding HRs associated with continuous testosterone levels within the three BMI classes are plotted in Fig. 2 considering the first quartile of circulating testosterone (0.278 ng/mL) as reference value. Accordingly, Table 2 reports for each BMI category, the numerical values of HR plotted in Fig. 2, for three specific values of testosterone level: 0.403 ng/mL (corresponding to the median), 0.532 ng/mL (corresponding to the third quartile) and 0.782 ng/mL (corresponding to the 95% percentile), with respect to 0.278 ng/mL, which corresponds to the first quartile of testosterone.

**Table 2:** Hazard ratio (HR) of relapse of ER-positive postmenopausal breast cancer patients for median (0.403 ng/mL), third quartile (0.532 ng/mL) and 95% percentile (0.782 ng/mL) with respect to the first quartile (0.278 ng/mL) of testosterone levels, according to BMI groups

Testosterone^a^ level (ng/mL)	BMI < 25	BMI 25–30	BMI ≥30
	HR^b^ (95% CI)	HR^b^ (95% CI)	HR^b^ (95% CI)
0.403 versus 0.278	5.52 (1.65–18.49)	1.64 (0.69–3.91)	0.68 (0.40–1.14)
0.532 versus 0.278	4.55 (1.09–18.98)	2.45 (0.75–8.02)	0.61 (0.31–1.20)
0.782 versus 0.278	0.25 (0.01–6.55)	4.68 (1.39–15.70)	0.75 (0.22–2.55)

^a^Testosterone levels were included as a continuous variable into the model

^b^Tumour size and the number of metastatic lymph nodes were included in the Cox proportional hazard regression model as adjusting covariates. In parenthesis, pertinent 95% confidence interval

In normal-weight women, the risk of relapse showed a bell-shape course with higher risk of relapse for levels of testosterone around the median value and lower risk of relapse for low and high testosterone levels (Fig. 2). Women with a testosterone concentration around the median value (0.403 ng/mL) or third quartile (0.532 ng/mL) had high and significant HR of relapse (5.52; 95% CI: 1.65–18.49 and 4.55; 95% CI: 1.09–18.98, respectively), compared to women with a low testosterone concentration (0.278 ng/mL, first quartile, Table 2), whereas the estimation of the HRs for testosterone concentration higher than third quartile showed decrease/stabilization of HRs with a wide statistical uncertainty (Fig. 2 and Table 2).

In overweight patients the risk of relapse increased (1.64; 95% CI: 0.69–3.91, 2.45; 95% CI: 0.75–8.02 and 4.68; 95% CI: 1.39–15.70) at increasing levels of testosterone (0.403 ng/mL, 0.523 ng/mL and 0.782 ng/mL, respectively versus 0.278 ng/mL) (Fig. 2 and Table 2).

On the contrary, an inverse relationship between testosterone levels and risk of relapse was found in obese patients. In this BMI group, there was a non-significant trend towards decrease in the risk of relapse at increasing levels of testosterone (Fig. 2 and Table 2).

## Discussion

In the present study we examined the prognostic role of circulating testosterone levels, according to BMI as a proxy of adiposity, in a cohort of postmenopausal patients with ER-positive breast cancer.

Our main findings confirmed that serum testosterone measured at diagnosis is an important prognostic factor for ER-positive breast cancer [9–11]. The novelty of this work resides however in the demonstration of a different impact of circulating testosterone on the risk of relapse, depending on the BMI categories.

When we analyzed the relationship between circulating testosterone, BMI, and risk of relapse we observed an interesting and statistically significant interaction. Although our data showed a certain degree of statistical uncertainty due to sample size, we found that in normal-weight and overweight patients the HRs increased with increasing testosterone levels until about the median value. Thereafter, the two BMI categories showed a rather different behavior: overweight patients had a further increase in the hazard of relapse as testosterone increased, whereas according to the confidence intervals, normal-weight patients showed no evidence of increase, but a decrease/stabilization of HRs for higher testosterone levels. Conversely, obese patients appeared to be characterized by a risk of relapse that decreased when testosterone levels increased, suggesting that the presence of high testosterone levels could reduce the high risk of recurrence typical of obese women. The observation that high levels of testosterone are associated with high HRs of relapse in normal-weight and overweight women, but not in obese patients, raises interesting questions about the relationship between adiposity, circulating testosterone and endocrine treatment of breast cancer.

From a mechanistic point of view, the influence of testosterone on tumour growth is presumably different in the onset compared to the progression of the disease. While in the onset of breast cancer the androgenic conversion into estrogen is the most plausible mechanism involved in tumour growth by estrogen/ER signalling [33, 34], the same path is unlikely in disease progression. According to international guidelines for breast cancer management [31], most of the ER-positive patients are submitted to hormone treatment aimed to block the ER-mediated proliferative events when tamoxifen is given, or to inhibit androgen-to-estrogen conversion [34, 35] when aromatase inhibitors are used. It is, therefore, conceivable that in ER-positive normal-weight or overweight patients undergoing endocrine treatment, the high levels of testosterone mainly affect tumour relapse through an alternative estrogen/ER signaling, likely by androgen/AR proliferative axis or by favoring proliferation along growth factors pathway [35, 36]. Although our study is too small to provide association between high levels of circulating testosterone, AR status, BMI and prognosis, recent preclinical researches showed that AR-overexpression is involved in the mechanism of resistance to endocrine-therapy either with tamoxifen [37, 38] or aromatase inhibitors [39, 40]. In a tumour environment depleted of estrogen or where estrogenic action is inhibited, AR-overexpressing breast cancer cells may acquire alternative proliferation mechanisms through AR-dependent intracellular ER-signalling [37–40]. Interestingly, in the group of our ER-positive TPM-patients characterized by BMI < 25, no AR expression (18 patients) or testosterone levels lower than the first quartile value (< 0.278 ng/mL, 56 patients), no relapse was observed (data not shown). This observation is consistent with the above hypothesis, i.e., in the absence of AR or in the presence of very low testosterone levels, the androgen-driven resistance mechanism would not trigger.

A further pathway of drug-resistance influenced by increased androgenic activity and able to replace the estrogen/ER proliferative pathway may occur through the insulin/IGFI axis [35, 36]. In fact, in presence of abdominal fatness, high testosterone affects, in a mutual interaction, the development of insulin resistance, a pathological condition linked to hyperinsulinemia and IGF-I over-production, especially in a state of estrogen deficiency [18, 20, 41].

Regarding obese women, our data suggest that those with high testosterone have a lower risk of progression than obese women with low hormone levels. This last association did not reach statistical significance; anyway the interaction found between testosterone levels and BMI classes indicates that testosterone affect positively the prognosis of obese women compared to that of normal-weight or overweight patients. To our knowledge, studies investigating this issue and with which to compare our findings are lacking, and we believe that these data deserve further in-depth studies on a larger number of patients.

A limitation of the present study is that patients were not stratified by type of endocrine treatment (i.e. tamoxifen or aromatase inhibitor, AI). However, tamoxifen and AI are expected either to create a tumour environment poor in estrogen or to inactivate ER, thus blocking almost completely the functioning of estrogen/ER axis [35, 36]. Therefore, under both treatments, high testosterone levels may be involved in the activation of alternative estrogen/ER-signalling, through androgen-AR and/or growth factors pathways. The common denominator is that, regardless of the drug used, high testosterone levels may play an important role in the drug resistance onset, ultimately leading to disease progression [37–40]. Nevertheless, aware of these implications, we are planning to investigate this specific point in forthcoming studies with expanded TPM-cohort sample size.

## Conclusion

While the present findings need to be validated and further refined according to the type of endocrine treatment received, they suggest that an increased androgenic activity not only plays an important role in the onset of breast cancer as demonstrated consistently in the literature [3–5], but also impacts the prognosis of ER-positive breast cancer patients, according to patients adiposity. While increased testosterone levels are associated with worse prognosis in normal-weight and overweight patients, increased levels seemed to be associated with a better prognosis in obese patients, although the results did not reach statistical significance in the latter. Several mechanisms have been suggested for explaining the association between increased testosterone levels and worse prognosis [8, 33, 36–39]. The most probable is that it acts as an aromatase substrate for estrogen synthesis [25, 33, 34], but also may favor resistance to hormonal treatment through alternative ER-signaling [37–40], likely by AR-axis, and predisposing to the visceral fat accumulation and to the endocrine-metabolic imbalances associated with insulin resistance, which in turn mediate breast cancer growth [20–22].

Altogether, although the present results need to be further validated, they suggest that the evaluation of circulating levels of testosterone in association to BMI assessment at diagnosis may help to identify postmenopausal ER-positive patients at higher risk of relapse and potentially open new therapeutic strategies in breast cancer.

## Additional files

Additional file 1: **Figure S1.** Workflow for the selection of TPM-ER-positive postmenopausal breast cancer patients. Shows the workflow for the selection of ER-positive postmenopausal breast cancer patients, starting from the 592 initial women recruited consecutively in the TPM cohort from December 2003 to December 2006, at Fondazione IRCSS Istituto Nazionale Tumori of Milan. (PDF 42 kb)

Additional file 2: **Figure S2.** Boxplots of circulating level of testosterone (ng/mL) according to tumour histology (IDC = Invasive Ductal Carcinoma; ILC = Invasive Lobular Carcinoma), Grade (G), number of metastatic axillary lymph Nodes (N), progesterone receptor (PR) and HER-2 status of ER-positive postmenopausal breast cancer patients. Number and percentage of patients in each group are reported and *p*-values are given. The bar inside the box is the median value and the box upper and lower dimensions define the inter-quartile range. Shows the boxplots of circulating level of testosterone (ng/mL) according to the other tumour characteristics considered in the study (histology, tumour grade, axillary nodal status, PR and HER-2 status). On the whole, the results did not indicate an association between circulating level of testosterone and unfavorable tumour characteristics as high tumour grade, axillary involvement or HER2 overexpression. (PDF 22 kb)

## Abbreviation

AR: Androgen Receptor
BMI: body mass index
ER: Estrogen Receptor
HR: Hazard Ratio
HRT: Hormone-Replacement Therapy
IDC: Invasive Ductal Carcinoma
IGF-I: Insulin-Like Growth Factor-I
ILC: Invasive Lobular Carcinoma
INT: Fondazione IRCSS Istituto Nazionale Tumori of Milan
PR: Progesterone Receptor
SHBG: Sex Hormone-Binding Globulin
TPM: Testosterone Prognosis, Mammary cancer
