# Correction to: Observational study on the prognostic value of testosterone and adiposity in postmenopausal estrogen receptor positive breast cancer patients

**DOI:** 10.1186/s12885-018-4799-2

**Published:** 2018-09-10

**Authors:** Elisabetta Venturelli, Annalisa Orenti, Aline S. C. Fabricio, Giulia Garrone, Roberto Agresti, Biagio Paolini, Chiara Bonini, Massimo Gion, Franco Berrino, Christine Desmedt, Danila Coradini, Elia Biganzoli

**Affiliations:** 1 Department of Research, Fondazione IRCCS Istituto Nazionale dei Tumori, Via Venezian 1, 20133 Milan, Italy; 2 Laboratory of Medical Statistics and Epidemiology,“Giulio A. Maccacaro”, Department of Clinical Sciences and Community Health, University of Milan, Via Vanzetti 5, 20133 Milan, Italy; 3 Regional Center for Biomarkers, Department of Clinical Pathology and Transfusion Medicine, Azienda ULSS3 Serenissima, Regional Hospital, Campo SS Giovanni e Paolo 6777, 30122 Venice, Italy; 4 Breast Surgery Unit, Fondazione IRCCS Istituto Nazionale dei Tumori, Via Venezian 1, 20133 Milan, Italy; 5 Department of Diagnostic Pathology and Laboratory, Fondazione IRCCS Istituto Nazionale dei Tumori, Via Venezian 1, 20133 Milan, Italy; 6 Breast Cancer Translational Research Laboratory, J. C. Heuson, Institut Jules Bordet, Université Libre de Bruxelles, 121 Boulevard de Waterloo, 1000, Bruxelles, Brussels, Belgium; 7 Unit of Medical Statistics, Biometry and Bioinformatics, Campus Cascina Rosa, Fondazione IRCCS Istituto Nazionale Tumori, Via Vanzetti 5, 20133 Milan, Italy

## Correction

Following publication of the original article [1], the authors reported that the affiliation of author Annalisa Orenti was omitted

The missing affiliation number is 2, Laboratory of Medical Statistics and Epidemiology, “Giulio A. Maccacaro”, Department of Clinical Sciences and Community Health, University of Milan, Via Vanzetti 5, 20133 Milan, Italy, as shown on this Correction.
