# Supplementary material for: Observational study on the prognostic value of testosterone and adiposity in postmenopausal estrogen receptor positive breast cancer patients
Source: BMC Cancer. 2018 Jun 13;18:651. doi: 10.1186/s12885-018-4558-4 (PMC5998599; doi:10.1186/s12885-018-4558-4)
Supplement: Supplementary file 1 — Figure S1. Workflow for the selection of TPM-ER-positive postmenopausal breast cancer patients. Shows the workflow for the selection of ER-positive postmenopausal breast cancer patients, starting from the 592 initial women recruited consecutively in the TPM cohort from December 2003 to December 2006, at Fondazione IRCSS Istituto Nazionale Tumori of Milan. (PDF 42 kb) [file 12885_2018_4558_MOESM1_ESM.pdf]

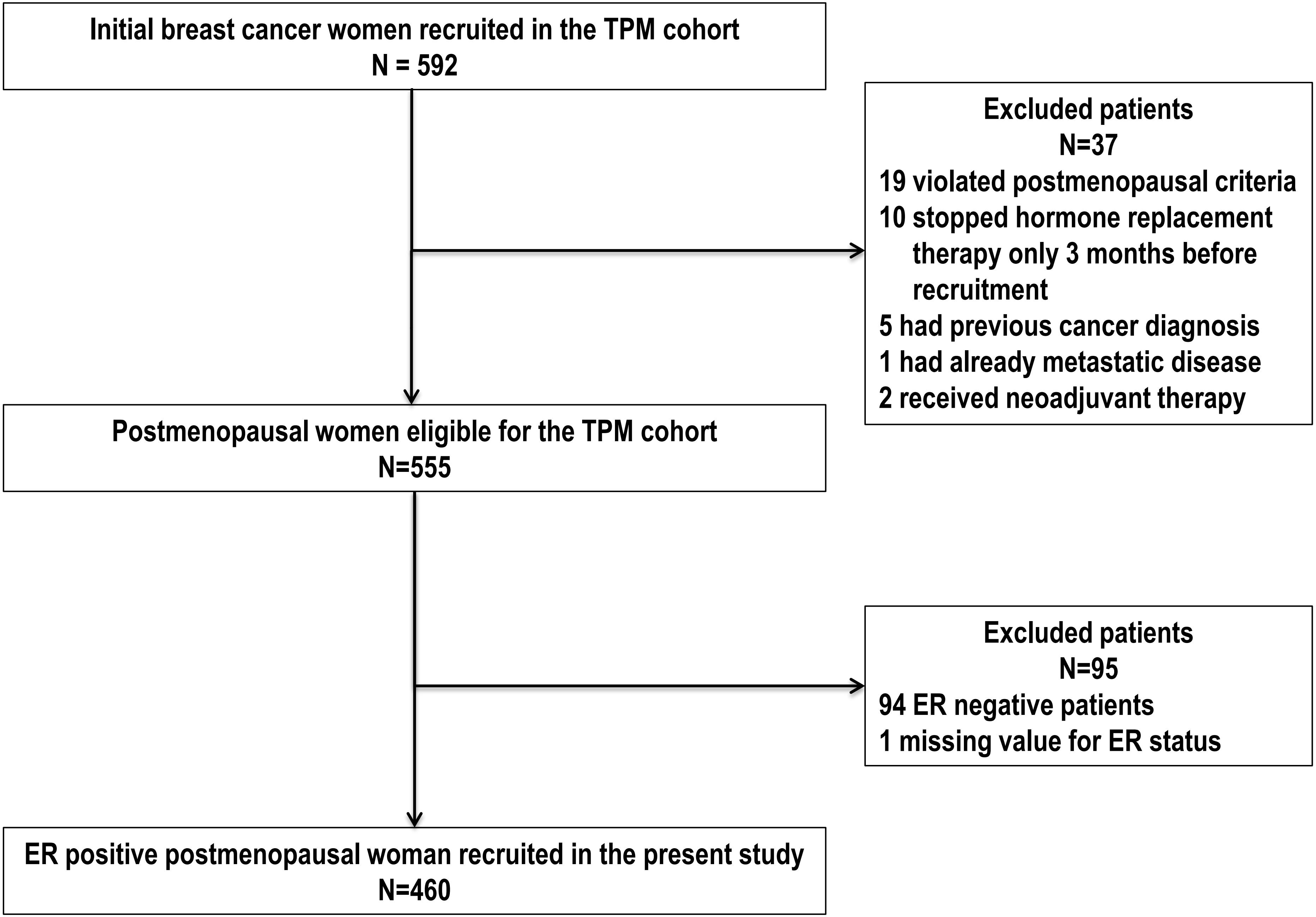
Initial breast cancer women recruited in the TPM cohort
N = 592
Excluded patients
N=37
19 violated postmenopausal criteria
10 stopped hormone replacement therapy only 3 months before recruitment
5 had previous cancer diagnosis
1 had already metastatic disease
2 received neoadjuvant therapy
Postmenopausal women eligible for the TPM cohort
N=555
Excluded patients
N=95
94 ER negative patients
1 missing value for ER status
ER positive postmenopausal woman recruited in the present study
N=460
