# Supplementary figures and images for: Observational study on the prognostic value of testosterone and adiposity in postmenopausal estrogen receptor positive breast cancer patients
Source: BMC Cancer. 2018 Jun 13;18:651. doi: 10.1186/s12885-018-4558-4 (PMC5998599; doi:10.1186/s12885-018-4558-4)

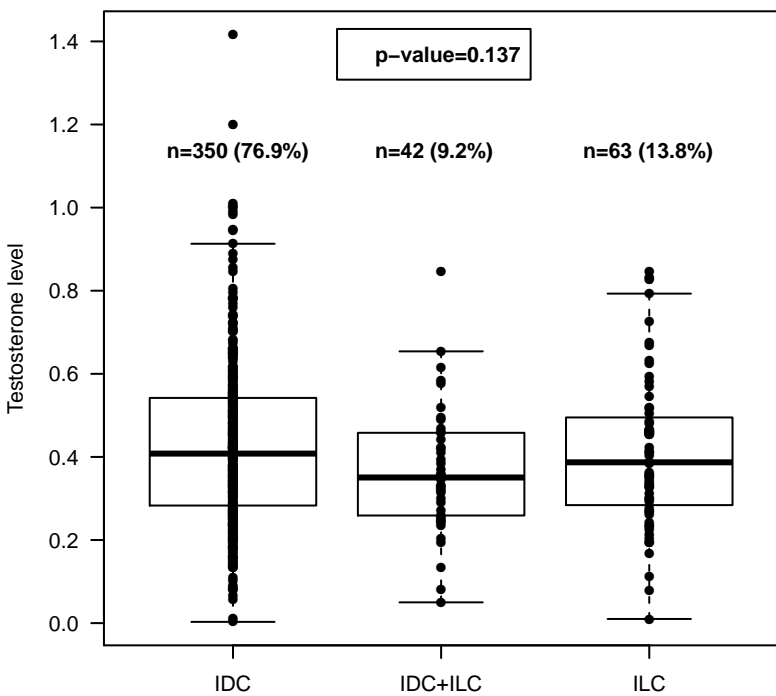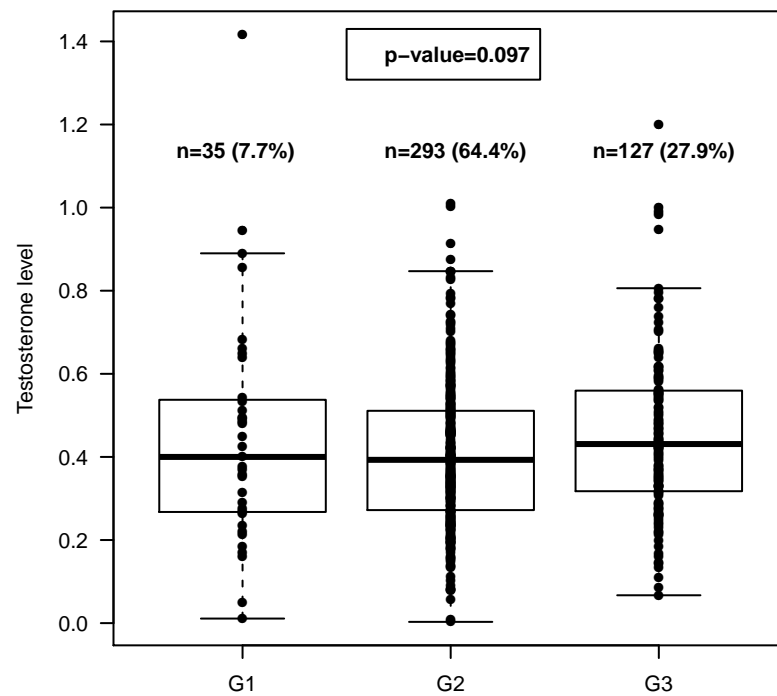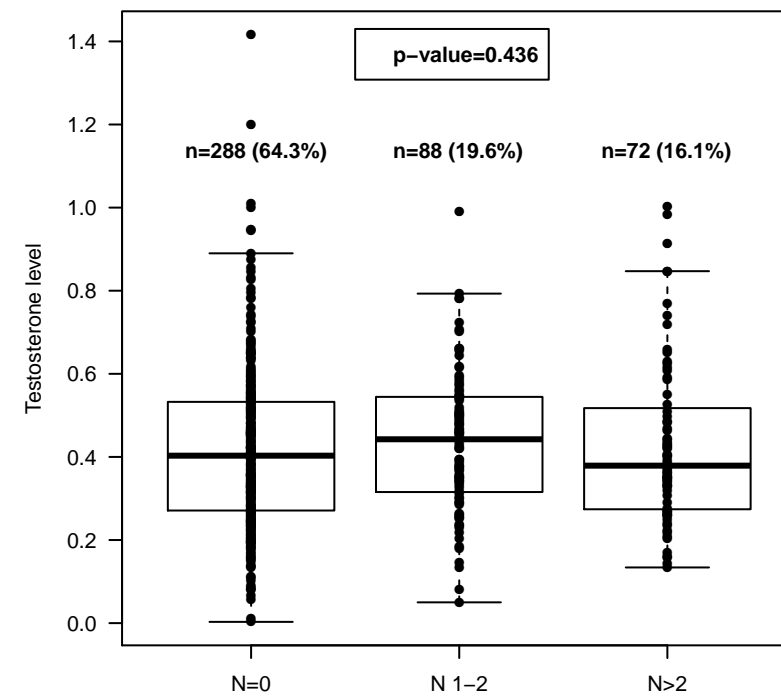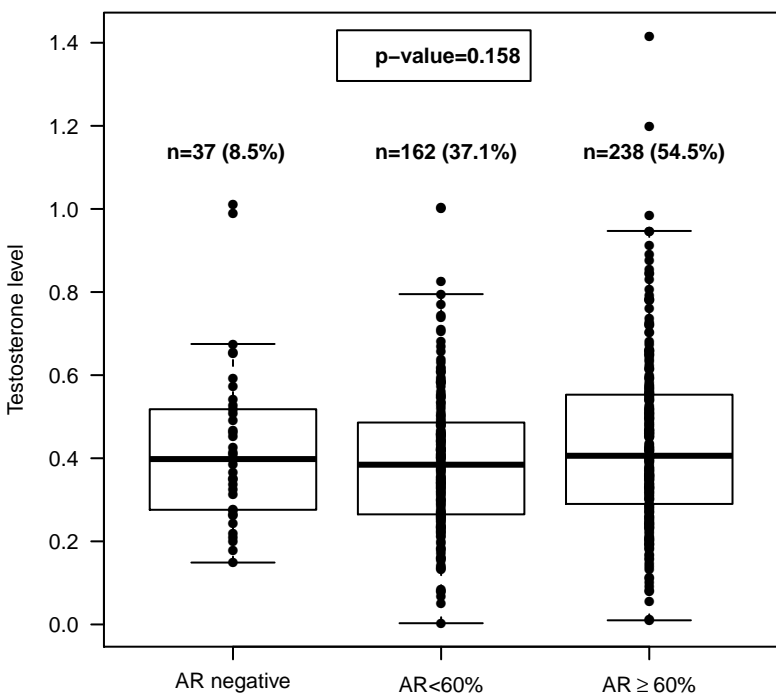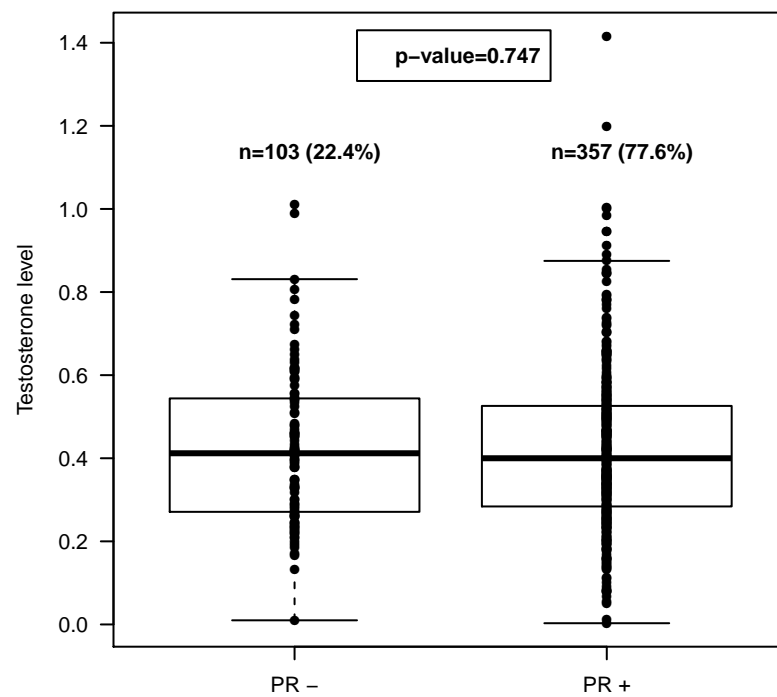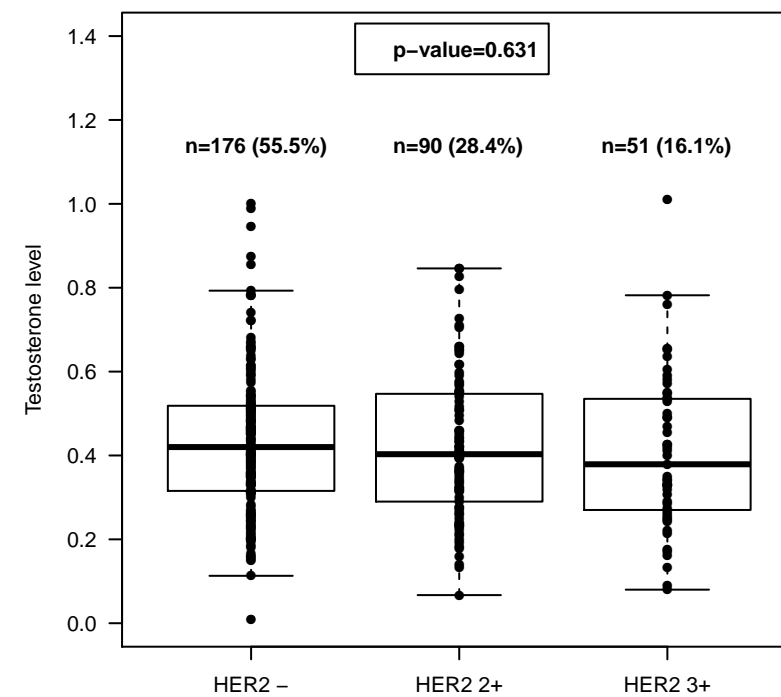

Supplement: Supplementary file 2 — Figure S2. Boxplots of circulating level of testosterone (ng/mL) according to tumour histology (IDC = Invasive Ductal Carcinoma; ILC = Invasive Lobular Carcinoma), Grade (G), number of metastatic axillary lymph Nodes (N), progesterone receptor (PR) and HER-2 status of ER-positive postmenopausal breast cancer patients. Number and percentage of patients in each group are reported and p-values are given. The bar inside the box is the median value and the box upper and lower dimensions define the inter-quartile range. Shows the boxplots of circulating level of testosterone (ng/mL) according to the other tumour characteristics considered in the study (histology, tumour grade, axillary nodal status, PR and HER-2 status). On the whole, the results did not indicate an association between circulating level of testosterone and unfavorable tumour characteristics as high tumour grade, axillary involvement or HER2 overexpression. (PDF 22 kb) [file 12885_2018_4558_MOESM2_ESM.pdf]
